# Impact of *Ocimum gratissimum*‐Supplemented Diet on Scopolamine‐Induced Cholinergic Disruption, Neuroinflammation, and Alzheimer's Disease‐like Memory Impairment in Swiss Mice

**DOI:** 10.1002/alz70859_096958

**Published:** 2025-12-25

**Authors:** Damilare Alabi Akanbi

**Affiliations:** ^1^ University of Ibadan, Ibadan, Oyo Nigeria

## Abstract

**Background:**

According to the World Health Organization, neurodegenerative diseases are on the rise. There are at least three factors that characterizes Alzheimer's disease: (i) a progressive loss of neuron function; (ii) the disease's molecular basis, which involves metalloprotein misfolding and aggregation (amyloid beta) or metal ion imbalances that cause oxidative stress; and (iii) the absence of a treatment to slow or stop the progression of symptoms at this time. Ocimum gratissimum has been shown to have neuroprotective effect due to wide range of bioactive compounds such as flavonoids and polyphenols. This study investigated the neuroprotective potential of Ocimum gratissimum‐supplemented diet on Scopolamine‐induced Alzheimers disease‐like memory impairment.

**Method:**

Thirty‐six adult male mice were randomly assigned to 6 groups (n=6). Group 1 received normal feed and water. Group 2 received Scopolamine (2 mg/kg) intraperitoneally for 14 days. Group 3 received the standard drug Donepezil (5 mg/kg) plus Scopolamine. Groups 4‐6 received Ocimum gratissimum‐supplemented diet (5%, 10%, 20%) plus Scopolamine. Neurobehavioral tests were conducted to assess spatial memory and navigation. The animals were then sacrificed, and biochemical analysis was performed to measure acetylcholinesterase activity, neuroinflammation and oxidative stress in the prefrontal cortex and hippocampus.

**Result:**

The Ocimum gratissimum‐supplemented diet groups showed significantly improved spatial memory and navigation compared to the Scopolamine‐only group, as evidenced by reduced transfer latency and increased discrimination index in the behavioral tests. Acetylcholinesterase activity was also significantly elevated in the prefrontal cortex and hippocampus of the Scopolamine‐treated mice.

**Conclusion:**

Ocimum gratissimum supplementation was able to improve spatial memory and protect against memory impairment, suggesting its potential as a neuroprotective agent against Alzheimer's disease‐like pathology.

